# Effect of the Use of Tomato Pomace on Feeding and Performance of Lactating Goats

**DOI:** 10.3390/ani10091574

**Published:** 2020-09-03

**Authors:** Waldeana C. F. Mizael, Roberto Germano Costa, George Rodrigo Beltrão Cruz, Francisco Fernando Ramos de Carvalho, Neila Lidiany Ribeiro, Aécio Lima, Rubén Domínguez, José M. Lorenzo

**Affiliations:** 1 Departamento de Zootecnia, Universidade Federal da Paraiba, CCA, Areia 58397-000, Paraíba, Brazil; dianamizael@yahoo.com.br; 2 Departamento de Ciência Animal, Universidade Federal da Paraíba, CCHSA, Bananeiras 58220-000, Paraíba, Brazil; betogermano@hotmail.com (R.G.C.); georgebeltrao@hotmail.com (G.R.B.C.); aeciomelolima@hotmail.com (A.L.); 3 Departamento de Zootecnia, Universidade Federal Rural de Pernambuco, Recife 52171-900, Pernambuco, Brazil; ffrcarvalho@hotmail.com; 4 Instituto Nacional do Semiárido–INSA, Campina Grande 58434-700, Paraíba, Brazil; neila.ribeiro@insa.gov.br; 5 Centro Tecnológico de la Carne de Galicia, Rúa Galicia No 4, Parque Tecnológico de Galicia, San Cibrao das Viñas, 32900 Ourense, Spain; rubendominguez@ceteca.net; 6 Área de Tecnología de los Alimentos, Facultad de Ciencias de Ourense, Universidad de Vigo, 32004 Ourense, Spain

**Keywords:** alternative feed, agro-industrial waste, goat diet, milk composition, *Solanum lycopersicum*

## Abstract

**Simple Summary:**

Inclusion of agro-industrial wastes reduces animal feed costs. However, it is very important to assess the effect this can have on the health and physiological condition of animals, as well as on the production and quality of milk. Therefore, this study was proposed with the objective of evaluating these aspects and verifying the optimal dose to maximize the farmers’ economic margin without affecting animals or their production. Inclusion of 40% tomato pomace does not influence the physiological characteristics of animals, and in turn improves both milk production and composition. Therefore, it can be concluded that formulation of the goats’ diet including 40% tomato pomace is the best option for animal diet.

**Abstract:**

The aim of this research was to evaluate the effect of including different levels of tomato pomace (TP) on performance, blood biochemical parameters, hormones, production and composition of milk, and economic analysis of *Saanen* goats reared in confinement. Sixteen multiparous goats (*Saanen*), 21 days in milk, were randomly distributed in two Latin square 4 × 4 (four periods and four treatments), according to the inclusion levels of dehydrated tomato pomace (0%, 20%, 40%, and 60%) in the diet. This inclusion resulted in differences in the intake of dry and organic matter, as well as ether extract, crude protein, water, neutral detergent fiber, and non-fibrous carbohydrates. The inclusion of 60% TP resulted in a significant decrease of body weight (−4.42 kg) in comparison with initial body weight, while the other three treatments did not affect or increase the animal body weight (between −0.05 and +3.07 kg). The addition of 20% and 40% of TP resulted in higher milk production (around 1.5 kg day^−1^) than in animals from a control (1.2 kg day^−1^) and 60% TP (1.04 kg day^−1^). This increase was approximately 28% in the animals with 40% of TP inclusion. Moreover, the addition of 20% or 40% TP also improved the milk quality, which presented a higher fat amount (4.37% and 4.63% in 20% TP and 40% TP animals, respectively) than in a control (3.7%) and animals feed with 60% TP (4.02%). The feed efficiency and feed conversion did not show differences between diets. Thyroid hormones (T3 and T4) were also significantly affected by the inclusion of TP in the diet. The diet with the highest level of TP (60%) had the lowest cost per kilo among the diets evaluated. However, the use of 40% TP in animal diet presented the highest milk production and intermediate production cost.

## 1. Introduction

In developing countries, ruminant production is limited by poor quality and scarcity of pastures, especially during periods of drought. Therefore, breeders are forced to use cereal-based concentrates. In recent years, the cost of concentrate used in animals feed has steadily increased, making the intensive farming systems more expensive [1]. The agricultural wastes and agro-industrial by-products of local origin are gaining renewed interest as alternatives to reduce feeding costs of ruminants [2,3], without changing the yield and quality of products of animal origin [4,5,6]. Additionally, environmental issues associated with both livestock production and by-products accumulation could be prevented [7].

The industrial tomato pomace (TP) stands out among the by-products of the fruit industry, being composed of seed, peel, and a small portion of pulp [8,9]. In the industries producing tomato juice and pulp, 5% to 10% of the tomato weight is considered as waste. TP has about 16% crude protein; 57% neutral detergent fiber; 44% fiber in acid detergent; 8% ether extract, and 24% lignin [10]. However, other authors reported higher amounts of waste during tomato processing. In this case, some steps (pasteurization at 80 °C, grinding and pressing) result in 20% to 42% of the weight of the fruit in waste, varying according to the type of processing used, enabling its use in animal production [11].

Due to its chemical composition and good acceptability by animals, tomato pomace has been used as an ingredient in the diet of small ruminants [3,12,13,14]. Studies have recently been carried out using tomato residue in the form of silage [15] and considering tomatoes together with other industrial residues [4] in a goat diet. The researchers concluded that these diets do not interfere with animal performance, milk production, and quality. However, studies evaluating the effect of diets using only tomato pomace as a single or bulky concentrate are scarce for dairy goats. Moreover, in this type of research they focus the studies exclusively on the nutritional and animal feeding aspects, but there are very few studies that carry out an economic analysis of the costs and the profit margins with the reformulation of the animal diets. Therefore, in the present manuscript we linked production costs with the amount and quality of the milk obtained, in order to have a global vision of both economic and productive aspects.

The hypothesis of our study is that the tomato residue, due to the characteristics of its composition, can replace forage in the goats’ diet, reducing the costs with feeding, without changing the performance of the animal. Therefore, the objective of the present research was to evaluate the effect of including different levels of tomato pomace on performance, blood biochemical parameters, hormones, production and composition of milk, and economic analysis in dairy goats kept in a confinement system.

## 2. Materials and Methods 

### 2.1. Animals, Diets, and Management

This study was approved by the Animal Ethics Committee of the Federal University of Paraíba (UFPB), Brazil (protocol no. 88/15), and it was conducted at the Federal University in Paraíba State (UFPB)-Campus at Bananeiras. The city is included in the geographical area covered by the Brazilian semiarid region at coordinates 6° 46′ S, and 35° 38′ W, with an altitude of 617 m. 

Sixteen *Saanen* dairy goats were used, all from the third and fourth lactation, with an average production of 1.31 ± 0.46 kg milk day^−1^ and with an average body weight of 46.2 ± 7.50 kg. Later, they were housed in individual stalls (1.50 m^2^), with free access to feed and water. The animals were kept in a confinement system for 68 days (four periods of 17 days). Each period consisted of 14 days of adaptation of the animals to the experimental diets and the last three days to collect data and samples (feces, feed, and milk).

Tifton 85 hay (*Cynodon dactylon* L.) was substituted for tomato pomace (TP) at levels of 0%, 20%, 40%, and 60% in the dry matter of the diets that contained soybean meal, corn, and a vitamin and mineral supplement (Table 1). The goats were randomly distributed in a Latin square (4 × 4), according to the inclusion levels of the diet. The diets were adjusted to meet the needs of lactating goats, producing 2.0 kg of milk day^−1^ and 4% fat [16], with a forage:concentrate ratio of 60:40. The feed offered and the leftovers were weighed to calculate the voluntary intake, and were adjusted daily, maintaining the leftovers at 10% based on the intake of the previous day.

The samples of the diets taken from the piles were immediately frozen in a refrigerator for further analysis. They were thawed and pre-dried in forced air at 55 °C for 72 h and was ground to a mesh size of a 1-mm sieve knife mill and packed into plastic bags. The determined nutrient content according to the Association of Official Analytical Chemists procedures [17] of ingredients and diets are shown in Table 1. The dry matter (DM; AOAC method 934.01), crude protein (CP; Kjeldahl method, AOAC method 984.13), ether extract (EE; AOAC method 920.39), neutral detergent fiber (NDF; method AOAC 973.18), and acid detergent fiber (ADF; AOAC method 973.18) were analyzed. Total carbohydrates were analyzed by capillary electrophoresis with ultraviolet radiation and derivatization pre-column with 250 mmol L^−1^ p-aminobenzoic acid (PABA) and 20% of acetic acid at 40 °C. Non-fibrous carbohydrate (NFC, %) was estimated using the equation proposed by Mertens [18]: NFC = 100 − (%CP + %EE + %DM+ %NDF). 

The collection of stool samples was performed directly in the rectal ampoule of the animals, daily (0, 2, 4, 6, 8, and 10 h after feeding), during the collection period. The samples were weighed, identified, and stored at −15 °C and at the end of the collection period, were homogenized (constituting a composite sample of animals) and pre-dried in an oven with forced circulation at 65 °C for 72 h. Indigestible NDF (iNDF) was used as an internal marker to estimate apparent nutrient digestibility and fecal output. An adult cannulated cow in the rumen was used. The animal received *ad libitum* feed, with 60% of Tifton hay, 20% of pomace waste, and 20% of concentrate (corn grain, soybean meal, mineral salt). For iNDF analysis, 0.5 g (1 mm) of feces, orts, and feed samples were in situ fermented (144 h) in the rumen of the cow in nylon bags. After ruminal incubation, the filter bags were washed and dried (temperature 55–60 °C for 72 h) and the incubation residues were analyzed for NDF concentrations. Fecal output was calculated by using the following equation: FE = iNDFI/iNDFF, in which: FE is the fecal output (kg/day); iNDFI is the iNDFI intake (kg/day); and iNDFFis the iNDF content in the feces (kg/kg).

Estimation of total digestible nutrients (TDN) was based on the equation described by Weiss [19]: TDN = CPD + EED × 2.25 + NFCD + NDFcpD. In this equation, CPD = (CP ingested − CP feces), EED = (EE ingested – EE feces), NFCD = (NFC ingested − NFC feces), and NDFcpD = (NDFcp ingested − NDFcp feces), is nutrient digestibility (g/Kg DM). In order to calculate metabolizable energy (ME) (kcal ME kg DM^−1^), the digestible energy (DE) was initially calculated as the product between total digestive nutrient (TDN) content and the factor 4.409/100, considering the ME concentration of 82% of DE [20].

### 2.2. Milk Sampling and Analysis 

Milking was performed manually, during the whole experiment period, occurring twice a day (06:00 and 15:00 h), including periods of adaptation and data collection, and the milk control was performed by individual weighing of the milk (kg day^−1^), during the three days of data collection of each period (every experimental period) respecting the proportion of milk milked (morning 70% and late 30%). Before milking, the goats’ udders were washed with chlorinated water and dried with paper towels and then tested for mastitis (black bottom mug test). After each milking was done post-dipping, the goats’ roofs were dipped in a 2% iodine solution. The weight of the animals was measured at the beginning and at the end of each data collection period. 

The vials and glassware were sanitized with distilled water and sterilized in an oven at 105 °C, in order to avoid contamination by milk residues from the previous milking. Samples from the morning production were conditioned in a refrigerated environment and later mixed with milk samples from milking in the afternoon, forming a sample composed of goat day. From the total milk milked per animal (kg day^−1^), 200 mL aliquot was withdrawn (the participation of the samples proportional to morning and afternoon milking) to analyze the physicochemical characteristics. After being conditioned in identified plastic bottles, the samples were submitted to a slow pasteurization thermal process at 65 °C for 30 min [21] and finally frozen at −4 °C for further analysis. The chemical analysis of fat (%), total solids (%), proteins (%), lactose (%), urea (mg dL^−1^), and casein (g 100^−1^) were performed using an Analyzer of Master Complete^®^ Milk (AKSO^®^, São Leopoldo, Rio Grande do Sul, Brazil), under specific technical conditions. 

### 2.3. Blood Biochemical Parameters 

Once the experiment diet had been introduced, blood samples were collected every two weeks, approximately 3 h after feeding. A total of three blood samples were taken [22]. To collect blood samples, the jugular vein was punctured after disinfection with iodine alcohol. For analysis of biochemical and hormonal parameters, blood was collected in 7 mL vacuum tubes containing separating gel and sodium fluoride (used for glucose analysis). The samples were homogenized, refrigerated, and taken to the laboratory for processing. All samples were centrifuged at 1100× *g* and 4 °C for 15 min. After centrifugation, the supernatants were collected and separated into 1.5 mL aliquots for biochemical and hormonal tests. Samples were stored at −20 °C until analysis [23], which was performed on the day following collection. A UV-Vis spectrophotometer (Thermo Scientific GENESYS 10S Vis, USA) was used to measure the following biochemical parameters: Total protein (TP), albumin (ALB), globulin (TP-ALB), glucose (GLU), triglycerides (TRI), cholesterol (CHO), and urea (URE). All tests were performed by using commercially available kits (Labtest, Lagoa Santa, MG, Brazil).

The plasma concentrations of total thyroxine (T4) and total triiodothyronine (T3) were measured in duplicate using an xMarkTM Microplate Absorbance Spectrophotometer (BIO-RAD, Hercules, CA, USA) and quantified by the enzyme linked immunosorbent assay (ELISA by competition) using a commercial kit (In Vitro diagnostic Ltd., Belo Horizonte, MG, Brazil) developed for the quantitative analysis of hormones.

### 2.4. Economic Analysis 

To calculate the cost of the diets, ingredients were evaluated based on prices from July 2020, considering the average price in the region. The economic analysis reflects the feeding cost, because the cost of milk was fixed at 0.454 USD L^−1^. Tomato pomace, because it is an industrial by-product, was obtained free of charge, therefore its inclusion in the feed formulation resulted in a decrease of the cost of animal diets. The final costs of the diets were 0.355, 0.296, 0.236, and 0.176 USD kg^−1^ for 0%, 20%, 40%, and 60% inclusion of TP, respectively. Costs of facilities and labour were considered fixed. Variables used for the economic analysis were recommended by Lana et al. [24], as follows: Average gross income (AGI)—obtained by multiplying the milk production by the price per kg of milk; feeding costs (FC)—obtained by multiplying the total amount of feed intake by its respective price; net income over feed costs obtained by the difference between the average gross income and feeding cost.

### 2.5. Statistical Analysis 

The design adopted was a Latin square 4 × 4 (4 treatments (levels 0%, 20%, 40%, and 60% of TP) and four animals) with four periods. Using the following mathematical model: Yijk = μ + Ti + Vj + Pk + εijk, where Yijk is the observation of animal i, in period j, receiving treatment k; μ is the general average; Ti is the effect of treatment i, where i = 1, 2, 3, and 4; Vj is the effect of animal j, where j = 1, 2, 3, and 4; Pk is the effect of period k, where k = 1, 2, 3, and 4; εijk is the random error associated with each ijk observation.

The data were submitted to analysis of variance, using the PROC GLM of the SAS^®^ program, the treatment averages were compared by the Tukey test at the 5% level of significance and regression analysis using the PROC REG of the SAS^®^ program.

## 3. Results

The inclusion of tomato pomace (TP) in dairy goats diets had a significant effect (*p* < 0.05) on the intakes of all nutrients (Table 2).

DM, organic matter, EE, CP, neutral detergent fiber, non-fibrous carbohydrates, and water intakes showed a linear increase up to the level of 40% of TP, and at the level of 60% TP the values of these variables decreased. The value of mineral material intake decreases as TP was included in the diet. The levels of inclusion of TP in the diet stimulated a quadratic behavior in the variables DM, organic matter, mineral material, EE, CP, neutral detergent fiber, non-fibrous carbohydrates, and water intakes. 

TP levels influenced the results of apparent digestibility of crude protein, ether extract, and non-fibrous carbohydrates, but there was no influence on the neutral detergent fiber digestibility (Table 2). The inclusion of TP resulted in a significant increase of these parameters, and the animals fed with 60% TP had the highest values of apparent digestibility of ether extract and non-fibrous carbohydrates (similar values than those obtained from 40% TP diet), while the apparent digestibility of crude protein was significantly higher in all experimental diets in comparison with the animals fed with a commercial diet. 

**Figure 1:**
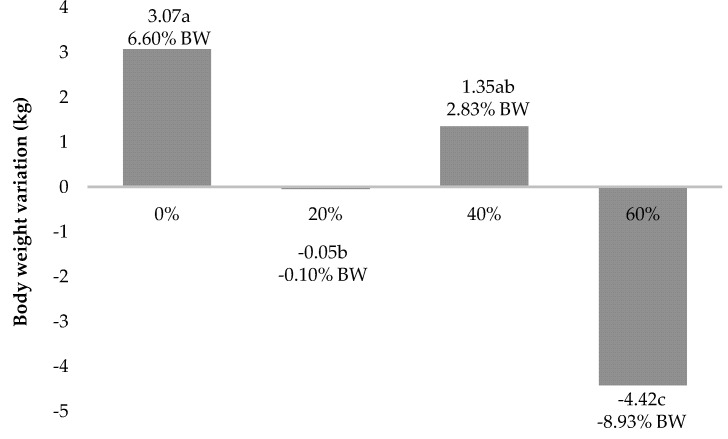
Body weight (BW) variation of lactating goats fed with different tomato pomace levels.

The final body weights showed a drop of more than 4 kg in the treatment animals with the inclusion of 60% of the tomato pomace. The goats fed with 20% TP remained at steady-state, while animals at the 0% and 40% TP level had increased their body weight (Figure 1).

The inclusion of TP had a significant effect on milk production (*p* < 0.05). This variable increased up to the level of 40% and in animals fed with 60% of TP the milk production decreased. With the addition of 20% and 40% TP, milk production increased linearly (*p* < 0.05). This increase represented an increase of approximately 28% in the diet with 40% of TP inclusion. However, in the diet with 60% TP, the milk production was 1.03 kg day^−1^, representing a drop of 33.7% and 15% in production when compared with the animals that received 40% and 0% of TP, respectively. Similarly, the milk production corrected to 4% of fat increased to the level of 40% TP (Table 3). The inclusion of TP had no significant effect (*p* > 0.05) on efficiency and feed conversion. 

The inclusion of TP in goat diets had no significant effect (*p* > 0.05) on the chemical constituents of milk, with the exception of lactose. Lactose showed the lowest value in milk from animals fed with 40% of inclusion of TP. Fat and lactose showed a quadratic effect, and for fat there was a growth up in milk from 40% TP group and a drop in the milk from 60% TP (Table 4).

The inclusion of TP in the diet of dairy goats did not influence blood biochemical parameters (glucose, cholesterol, urea, and albumin) and thyroid hormones (T3 and T4). Total protein and globulin had a linear effect, and T4 have a quadratic effect (Table 5).

**Figure 2:**
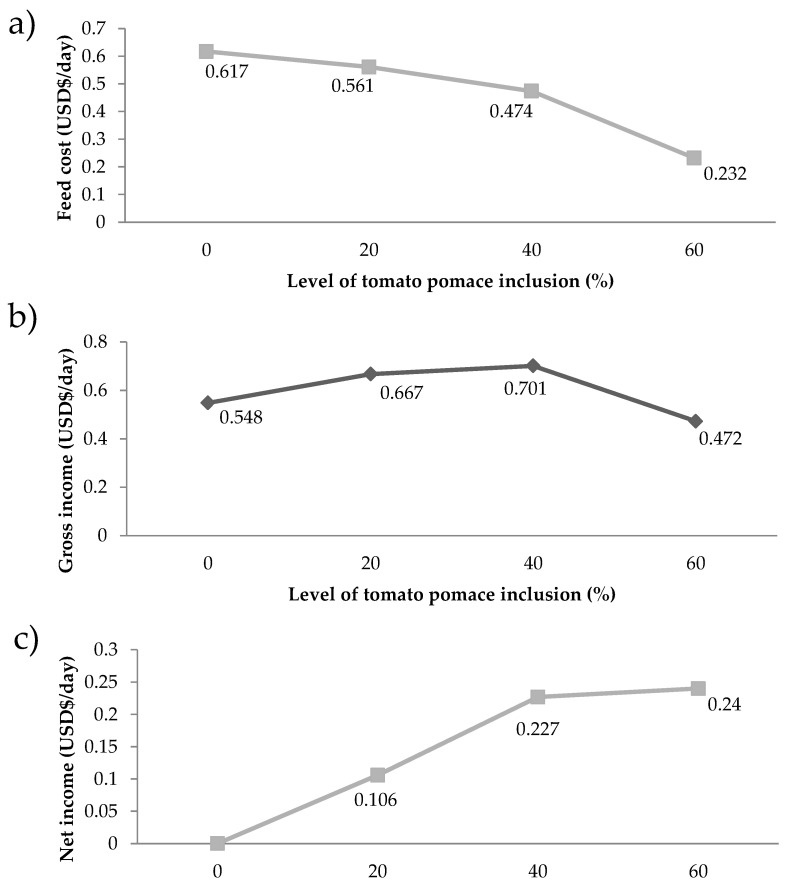
Feeding cost (**a**), gross income (**b**), and net income (**c**) of goat diets with different levels of tomato pomace.

The diet with 60% TP presented the lowest cost and the lowest milk production, among the other levels. The best milk production was obtained by including 40% TP, with an average cost of 0.236 USD kg^−1^. On the other hand, the diet without the inclusion of the tomato pomace presented the highest cost, showing the reduction in cost when incorporating tomato residue in the feed of goats. These results have a direct impact on feed costs, where intake and feed costs are taken into account. This low cost of feed provided the lowest income and net income (Figure 2).

## 4. Discussion

According to the requirements recommended by NRC [16], the average intake for a goat with a body weight of 40 kg, producing 2 kg of milk day^−1^ with a fat content of 3.5% is 1.80 kg DM day^−1^ and 4% to 5% body weight. This value is close to those found in treatments with 0%, 20%, and 40% of TP in the diet. Possibly the decrease in DM intake at the 60% TP level may be due to a reduction in the palatability of the diet. Another fact and, probably, what fits most is that although the feed formulation had a 42% neutral detergent fiber in the diet at the level of 60% TP, the form that the feed was processed did not take into account the value of the effective neutral detergent fiber, since the pomace was included in the meal in a bran form along with the other ingredients that were also provided in the form of bran. Romero-Huelva et al. [1] did not find significant differences in DM intake when goats were fed with a diet containing olive pomace and tomato residues (12% and 12.5% on a feed basis, respectively for olive pomace and tomato).

Fiber is necessary to stimulate chewing activity, maintain the flow of saliva, and a favorable ruminal environment for the development of microorganisms responsible for the digestion of fibrous carbohydrates [27]. In addition, it is necessary to take into account the importance of physical characteristics of the feed (particle size), conceptualized as physically effective fiber (peNDF). peNDF is responsible for increasing the secretion of buffers from saliva, which keeps the ruminal pH at an optimal level, which is directly related to animal health and preservation of milk fat. As a consequence of inadequate peNDF in the diet of ruminants, there is a decrease in rumen pH, a decrease in DM intake, a drop in milk fat content, and an increased risk of metabolic disorders, such as acidosis [28].

According to Ventura et al. [29], tomato residue can be used up to 100 g DM as feed for adult goats without suffering digestive disorders. The tomato residue provides 2.58 Mcal kg^−1^ DM of digestible energy and a small amount of available rumen protein (33 g kg DM^−1^) [30]. 

Up to the limit of 20% in the diet, the presence of TP improved the digestibility of crude protein. For ether extract and non-fibrous carbohydrates, digestibility improved up to the limit of 40%. This is a positive aspect of the use of TP, whose presence increases the energy of the diet and the content of ether extract, which reflected in the greater digestibility of this component and the greater amount of ether extract in milk (Table 3).

During the experimental period, three goats treated with 60% TP showed a sharp drop in performance, decreased milk production, and in one of the goats this condition evolved to anorexia, diarrhea, dehydration, ruminal atony, apathy, congested mucous membranes, and death. The clinical diagnosis was frothy bloat, according to the pathologist who performed the autopsy, and the cause of death was septic shock secondary to pre-stomach injuries. The necrotic lesions of the mucosa of the pre-stomach resulted from ruminal acidosis. In cases of ruminal acidosis, a low pH induces chemical damage to the mucous membranes [31]. These lesions allow bacteria to enter the organ wall and reach the bloodstream, resulting in septic shock. Pulmonary edema and renal infarctions are consequences of shock [32].

Abdollahzadeh et al. [33], who evaluated the inclusion of tomato + apple pulp (50/50) silage to replace alfalfa hay in the feeding of Holstein cows, observed a decrease in chewing activity and, as a consequence, a reduction in rumen pH and a higher concentration of acetic and propionic acids. The reduction in chewing resulted in a lower production of saliva that has the buffering function in the animal’s rumen [27]. The reduction in neutral detergent fiber degradation was also confirmed.

There is a negative correlation between the physically effective fiber (peNDF) and the ruminal pH. According to Abdollahzadeh et al. [33], the subclinical acidosis was observed at a pH value of 5.8, and peNDF is a reliable indicator of chewing activity. In addition, as commented above, peNDF is related to the time of ingestion x rumination, where a longer rumination time increases the buffering capacity of saliva and leads to an improvement in rumen pH [34], which is directly related to animal health and milk fat content [35]. Particular attention should be paid to the size of the particles as the inclusion of tomato residue in the diets increases. The diet with the highest inclusion of TP (60%) presented the largest amount of smaller particles among the diets.

The low DM intake may have reflected in the intake of some nutrients, since the composition of the diets in the proportion of CP, neutral detergent fiber, and total digestible nutrients did not show a significant variation between them, and the same statistical behavior for the intake result was presented for the DM intake. According to NRC [16], the required CP intake is 0.278 g day^−1^, which was not met at the maximum level of TP inclusion (60%). However, mineral material and EE intakes are linked to the composition of the diet. In the present study, it was observed that as the level of TP in the diet increased, the mineral material intake decreased, because the mineral sources are different in each treatment. 

According to NRC [16], for each 1 kg of DM ingested by lactating goats, 2.9 L of water (feed + drinker) are required per day. In this sense, the data from the present study showed that only the treatment with 20% of TP was in accordance with that recommendation.

Abdrollahzadeh [36] evaluated the effect of the inclusion of dried tomato pomace (10,% 20%, and 30%) on the diet of Markho goats. These authors confirmed that the carcass characteristics and blood metabolites, such as the glucose, total proteins, urea, cholesterol, and triglycerides did not show significant differences among groups. For this goat breed, the recommended level was 20% inclusion of dried tomato pomace.

In the present research, higher milk production and lower composition results were reported by Arco-Pérez et al. [37]. These authors evaluated the use of silage with olive oil and tomato residue in the feeding of dairy goats. They concluded that the use of tomato in goat diet did not influence milk production in comparison with animals that received a control diet (around 1.7 kg day^−1^). Abbeddou et al. [13] evaluated supplementary diets of sheep with tomato paste and observed the following values: 17.4%, 6.33%, 5.44%, and 5.17% for total solids, fat, protein, and lactose, respectively. Assessing the effect of including tomato residue + apple pulp silage on the performance of Holstein cows, it was observed that the group of animals that received 15% silage had the worst milk production among the levels studied (0%, 15%, and 30%) [33].

According to Arco-Pérez et al. [37], the dietary strategy of replacing oats hay in the diet of lactating goats with silages made of tomatoes or olive oil by-products together with SFO supplementation (20 g kg of DM^−1^) improved the milk quality without affecting animal efficiency. Furthermore, the long-term use of tomato silage in dairy goats fosters voluntary intake, which resulted in higher body weight gain, without compromising the milk production and composition. Such findings suggest that using silages made with tomato surplus in dairy goats is a feasible dietary strategy in areas where the by-product is available.

The chemical composition of the milk constituents was higher than those recommended by the current legislation in Brazil for goat’s milk [21], which comprises the values of 2.9 for fat, 11.1 for total solids, and 2.8 for protein. Lobo et al. [38] evaluated Alpine and Saanen in Northeastern goats and observed values for physicochemical parameters (fat = 3.76% and 3.24%; protein = 2.95% and 2.59%; lactose = 4.07% and 4.14%; and total solids = 11.89% and 11.05%, respectively) of milk similar to our research. Other authors reported lactose values of 4.30% in Saanen goat milk [39] from UK, while Olivier et al. [40] found in the same goat breed fat and protein amounts of 3.14% (South Africa) and values of 3.2%, 2.8%, and 4.2% for fat, protein, and lactose, respectively were reported in Saanen goats from Mexico [41]. Proportions of protein lower than 2.8% were observed by Clark and Sherbon [42] for Toggenburg and Alpine × Saanen crossbred reared in the United States of America. 

The inclusion of TP in the diet of goats significantly affected the lactose contents. Lactose is one of the most stable nutrients in the chemical composition of milk and is directly related to the regulation of osmotic pressure in order to extract water from the blood into the milk, so that higher lactose production determines greater milk production with the same content of lactose [43]. Another important point is that milk production in goats fed with a 60% TP diet was inversely proportional to lactose production. It was expected that in larger amounts of lactose the milk production would be higher, which did not happen, probably due to a low dry matter intake in this treatment (Table 2). 

The plasma glucose content is above the reference values (50 to 75 mg dL^−1^) mentioned by Kaneko et al. [44]. According to González et al. [43], plasma glucose is not a good indicator of the nutritional energy status resulting from the insensitivity of glycemia to nutritional changes and its sensitivity to stress. The regulation of this element occurs through homeostatic mechanisms that are quite efficient in the body, which involve the endocrine control by insulin and glucagon [45]. Thus, large variations in gluconeogenesis and glucose use do not necessarily result in considerable variations in the blood glucose level [46]. The cholesterol values above the reference values are 80 to 130 mg dL^−1^ [44]. There are no reference values for triglycerides in the goat species in the literature. However, when compared to other ruminants, such as sheep, these were found to be within the normal range (from 2 to 32 mg dL^−1^ for sheep) [44].

The plasma protein showed a slightly higher content than normal values for the goat species in all treatments. According to Kaneko et al. [44], this value ranged from 6.4 to 7.0 g dL^−1^. The decrease in total proteins is related to feed deficiency, when pathological causes are ruled out [43], which was not verified in the present study (since the total proteins remained slightly above the reference values). Serum albumin concentrations showed normal values for the goat species, whose reference values are 2.7 to 3.9 g dL^−1^, whereas globulin showed values above normal for the species, which is 2.7 at 4.1 g dL^−1^ [44].

The serum urea concentrations were above the values considered normal for the caprine species, which vary from 21.4 to 42.8 mg dL^−1^ [44]. Elevated plasma urea levels may be related to a lack of adequate balance between protein and energy levels in the diet, with a high protein intake or energy deficit, which would lead to a greater accumulation of ammonia in the rumen, with increased formation of urea by the liver [47]. In our study, the absence of significant differences in urea content among different diets demonstrates that the inclusion of tomatoes maintains the energy/protein balance, so it can be a good ingredient in the reformulation of diets. 

Thyroid hormones act in all tissues of the body and increase the basal metabolic rate, make more glucose available in cells, stimulate protein synthesis, and increase lipid metabolism [23]. According to the composition of the diets (Table 1), levels of ether extract increased with the inclusion of TP in the diet. Probably, this may have increased the metabolism of lipoproteins in the intestine and especially in the liver. However, it is important to note that there is no information in the literature on the effect of feed using TP in blood and hormonal parameters of goat. Costa et al. [22] used guava agro-industrial residue in the feed of confined sheep and observed that cholesterol and thyroid hormones (T3 and T4) was modified with the inclusion of this residue in the animal diet. Cholesterol showed values of 173; 158; 146; 144; and 146 mg dL^−1^, T3 was 6.98; 7.29; 7.99; 7.93; and 8.30 ng mL^−1^, whereas T4 presented concentration values of 19.3; 19.9; 20.3; 20.6; and 21.5 ng mL^−1^. 

The results of feed cost were favourable for the highest level of inclusion of tomato pomace. This is due to the fact that this diet had the lowest feed intake and its diet was the cheapest among those studied. As observed in Table 1, the lower cost of diets with increasing tomato pomace content is due not only to the use of this industrial by-product, but despite increasing the soybean meal content, the proportions of ground corn and Tifton hay decrease. Therefore, the price of each of the diets reflects all these variations. Taking into account the price of a liter of goat’s milk practiced in the region (USD 0.454 L^−1^) and already knowing the cost of feeding each diet, the best value for gross income were obtained for animals feed with 40% TP, while the gross margin was similar between the animals feed with 40% and 60% TP (between 0.227 and 0.24 USD day^−1^). Thus, in view of the economical results, the inclusion of 40% TP is the best option without affecting the animals health and improving milk production. As occurred in our study, Costa et al. [6] evaluated the use of guava agro-industrial by-product in the feed of confined sheep and observed that the inclusion of the by-product led to a reduction in feed costs. 

The inclusion of agro-industrial by-product is an opportunity to significantly improve the quality of milk and the profit margins of the farmer by lowering the price of animal feed. However, it is very important to set the best incorporation dose, since an unbalanced diet can lead to serious problems for animals. This was the case of the animals that were supplied with a diet with 60% TP. However, the other levels of tomato inclusion (20% and 40%) improved the milk production in goat without affecting the other parameters. In this regard, the inclusion of 40% of TP presented the best milk production and allowed reducing the feeding costs.

## 5. Conclusions

The inclusion of 40% TP presented the best results, so it can be affirmed that the formulation of the goat diet with 40% would be a good strategy to increase the benefits without affecting the physiological state of the animal. Additionally, animals that received 40% TP increased both the milk production and the quality of the milk these animals produced.

## Figures and Tables

**Table 1:** Percentage and chemical composition of experimental diets.

Ingredient (g kg^−1^ DM)	Levels of Tomato Pomace Inclusion (%)			
	0.00	20.0	40.0	60.0
Tomato industrial pomace (TP) ^1^	0.00	200	400	600
Tifton hay	600	400	200	0.00
Ground corn	220	285	335	385
Soybean meal	165	100	50.0	0.00
Mineral supplement ^2^	7.0	7.0	6.0	5.0
Calcitic limestone	4.0	6.0	9.0	10.0
Dicalcium phosphate	4.0	2.0	0.00	0.00
*Chemical composition*				
Dry matter, DM (g kg^−1^ as fed)	869	866	863	859
Crude protein, CP (g kg^−1^ DM)	146	141	142	143
Ether extract, EE (g kg^−1^ DM)	175	249	320	391
Neutral detergent fiber, NDF (g kg^−1^ DM)	549	510	470	430
Acid detergent fiber, ADF (g kg^−1^ DM)	279	293	306	319
Total Carbohydrates, TC (g kg^−1^ DM)	815	814	808	801
Non-fibrous carbohydrates, NFC (g kg^−1^ DM)	265	304	337	371
Metabolizable energy, ME (Mcal kg^−1^ DM)	2.38	2.49	2.59	2.70
Diet cost (USD kg^−1^) ^3^	0.355	0.296	0.236	0.176

^1^ DM: 84.30%, EE: 3.74%, NDF: 56.67%, ADF: 45.99%, NFC: 781.84 g, TC: 215.14 g; ^2^ composition of mineral supplement per kg: P: 70 g; Ca: 140 g; Na: 148 g; S: 12 g; Mg: 1.320 mg; F: 700 mg; Zn: 4.700 mg; Mn: 3.690 mg; Fe: 2.200 mg; Co: 140 mg; I: 61 mg; Se: 15 mg; Sodium monensin: 100 mg; ^3^ costs of the ingredients (USD kg^−1^): Tomato pomace: 0; Tifton hay: 0.301; Ground corn: 0.435; Soybean meal: 0.405; Mineral supplement: 1.372; Calcitic limestone: 0.125; Dicalcium phosphate: 0.505.

**Table 2:** Body weight and nutrient intake by dairy goats fed with different levels of inclusion of tomato pomace.

Variables	Levels of Tomato Pomace Inclusion (%)				SEM	*p-*Value		
	0.00	20.0	40.0	60.0		Tukey	Linear	Quadratic
Initial body weight (kg)	46.35	48.05	47.71	49.51	6.57	0.708	0.280	0.978
Final body weight (kg)	49.41	48.00	45.08	45.08	6.77	0.397	0.174	0.516
Nutrient intake								
Dry matter (kg day^−1^)	1.75 ^ab^	1.90 ^a^	2.00 ^a^	1.31 ^b^	0.41	0.001	0.050	0.001 ^1^
Organic matter (kg day^−1^)	1.42 ^a^	1.54 ^a^	1.62 ^a^	1.05 ^b^	0.38	0.001	0.037	0.001 ^2^
Mineral material (g day^−1^)	0.12 ^a^	0.10 ^a^	0.07 ^b^	0.03 ^c^	0.02	<0.0001	<0.0001	0.038 ^3^
Crude protein (g day^−1^)	0.26 ^ab^	0.28 ^a^	0.30 ^a^	0.21 ^b^	0.21	0.002	0.110	0.002 ^4^
Neutral detergent fiber (g day^−1^)	0.84 ^a^	0.88 ^a^	0.89 ^a^	0.56 ^b^	0.20	0.000	0.002	0.001 ^5^
Ether extract (g day^−1^)	0.04 ^c^	0.07 ^b^	0.09 ^a^	0.08 ^ab^	0.08	<0.0001	<0.0001	<0.000 ^6^
Non-fibrous carbohydrate (g day^−1^)	0.51 ^a^	0.56 ^a^	0.59 ^a^	0.39 ^b^	0.13	0.001	0.062	0.000 ^7^
Apparent digestibility (%)								
Crude protein	0.65 ^b^	0.73 ^a^	0.74 ^a^	0.74 ^a^	0.068	<0.0001	<0.0001	0.0002 ^8^
Ether Extract	0.61 ^c^	0.78 ^b^	0.83 ^a^	0.85 ^a^	0.013	<0.001	<0.0001	<0.0001 ^9^
Neutral Detergent Fiber	0.58	0.56	0.56	0.55	0.053	0.2828	0.0729	0.5398
Non-fibrous Carbohydrates	0.86 ^c^	0.92 ^b^	0.93 ^ab^	0.95 ^a^	0.052	<0.0001	<0.0001	0.0451 ^10^
Water (L day^−1^)	4.57 ^ab^	5.06 ^ab^	5.18 ^a^	3.80 ^b^	0.05	0.041	0.210	0.012 ^11^
IWDM (L kg DM^−1^)	2.63	2.74	2.63	2.91	0.64	0.682	0.360	0.611

IWDM: Water intake per kilogram of ingested dry matter; SEM: Standard error of the mean; ^a–c^ Mean values in the same row (corresponding to the same parameter) not followed by a common letter differ significantly (*p* < 0.05; Tukey test); ^1^ Y = 0.99 + 0.93x − 0.21 × ^2^ (R^2^ = 0.90); ^2^ Y = 0.82 + 0.74x − 0.16 × ^2^ (R^2^ = 0.90); ^3^ Y = 0.12 + 0.11x − 0.01 × ^2^ (R^2^ = 0.99); ^4^ Y = 0.14 + 0.13x − 0.02 × ^2^ (R^2^ = 0.86); ^5^ Y = 0.55 + 0.37 − 0.09 × ^2^ (R^2^ = 0.93); ^6^ Y = −0.02 + 0.07x − 0.01 × ^2^ (R^2^ = 0.94); ^7^ Y = 0.27 + 0.28x − 0.09 × ^2^ (R^2^ = 0.91); ^8^ Y = 0.65466 + 0.00439 X + 0.00005039 X^2^ (R^2^= 0.97); ^9^ Y = 0.61372 + 0.00962 X + 0.00009566 X^2^ (R^2^= 0.99); ^10^ Y = 0.87194 + 0.00241 X + 0.00001953 X^2^ (R^2^= 0.96); ^11^ Y = 0.11 + 0.12x − 0.02 × ^2^ (R^2^ =0.94).

**Table 3:** Milk production, feed efficiency, and feed conversion of dairy goats fed with different levels of inclusion of tomato pomace.

Variables	Levels of Tomato Pomace Inclusion (%)				SEM	*p-*Value		
	0.00	20.0	40.0	60.0		Tukey	Linear	Quadratic
Milk production (kg day^−1^)	1.20 ^ab^	1.47 ^ab^	1.54 ^a^	1.04 ^b^	0.43	0.022	0.502	0.003 ^1^
Milk production 4% (kg day^−1^) ^†^	1.13 ^b^	1.56 ^ab^	1.70 ^a^	1.03 ^b^	0.49	0.004	0.917	0.001 ^2^
Feed efficiency (kg kg^−1^)	0.68	0.77	0.77	0.80	0.15	0.238	0.066	0.459

^†^ Milk production corrected to 4% of fat; feed efficiency (milk production/dry matter intake); SEM: Standard error of the mean; ^ab^ mean values in the same row (corresponding to the same parameter) not followed by a common letter differ significantly (*p* < 0.05; Turkey test); ^1^ Y = 0.459 + 0.924x − 0.194x^2^ (R^2^ = 0.95); ^2^ Y= 0.020 + 1.357x − 0.274x^2^ (R^2^ = 0.95). The equation for the correction of milk production to 4% of fat (milk production 4% = (0.4 × kg of milk) + (15 × kg of milk fat))was proposed initially for cattle milk, however, it has also been shown to be effective for calculating goat’s milk and has been used by other authors [25,26].

**Table 4:** Chemical composition of milk from dairy goats fed with different levels of inclusion of tomato pomace.

Variables	Levels of Tomato Pomace Inclusion (%)				SEM	*p*-Value		
	0.00	20.0	40.0	60.0		Tukey	Linear	Quadratic
Fat (%)	3.70	4.37	4.63	4.02	1.00	0.136	0.348	0.032 ^1^
Total solids (%)	11.78	12.48	12.87	12.26	1.46	0.342	0.331	0.130
Protein (%)	2.93	3.03	3.20	3.00	0.53	0.652	0.581	0.331
Lactose (%)	4.17 ^ab^	4.09 ^ab^	3.97 ^b^	4.21 ^a^	0.22	0.045	0.927	0.014 ^2^
Urea (mg dL^−1^)	28.47	29.55	31.31	28.37	6.14	0.629	0.825	0.267
Casein (g 100^−1^)	2.31	2.46	2.62	2.41	0.51	0.520	0.460	0.232

SEM: Standard error of the mean; ^ab^ mean values in the same row (corresponding to the same parameter) not followed by a common letter differ significantly (*p* < 0.05; Turkey test); ^1^ Y = 2.283 + 1.720x − 0.319x^2^ (R^2^ = 0.97); ^2^ Y = 4.523 − 0.405x + 0.080x^2^ (R^2^ = 0.76).

**Table 5:** Blood biochemical parameters and hormones of dairy goats fed with different levels of inclusion of tomato pomace.

Variables	Levels of Tomato Pomace Inclusion (%)				SEM	*p-*Value		
	0.00	20.0	40.0	60.0		Tukey	Linear	Quadratic
Albumin (g dL^−1^)	3.11	3.51	3.17	2.81	0.70	0.163	0.231	0.077
Total protein (g dL^−1^)	7.23	7.40	7.88	7.71	0.59	0.544	0.016 ^1^	0.344
Globulin (g dL^−1^)	4.11	3.89	4.72	4.89	0.90	0.337	0.012 ^2^	0.448
Glucose (mg dL^−1^)	98.08	97.99	89.88	85.44	24.05	0.530	0.155	0.752
Cholesterol (mg dL^−1^)	137.21	136.19	132.90	147.32	22.26	0.487	0.420	0.250
Urea (mg dL^−1^)	87.09	83.00	94.27	84.93	16.25	0.365	0.768	0.620
Triglycerides (mg dL^−1^)	13.35	13.91	12.76	13.40	3.55	0.888	0.816	0.981
Hormones								
T3 (ng mL^−1^)	2.15	2.03	2.01	2.15	0.30	0.556	0.718	0.152
T4 (ng mL^−1^)	5.11	4.76	4.73	5.00	0.44	0.114	0.197	0.018 ^3^

SEM: Standard error of the mean; ^1^ Y = 7.26 + 0.01x (R^2^ = 0.71); ^2^ Y = 3.93 + 0.02x (R^2^ = 0.73); ^3^ Y = 5.11 − 0.03x + 0.0003x ^2^ (R^2^= 0.99).
